# Chemical Defense Mechanisms and Ecological Implications of Indo-Pacific Holothurians

**DOI:** 10.3390/molecules25204808

**Published:** 2020-10-19

**Authors:** Elham Kamyab, Sven Rohde, Matthias Y. Kellermann, Peter J. Schupp

**Affiliations:** 1 Institute for Chemistry and Biology of the Marine Environment (ICBM), Carl-von-Ossietzky University Oldenburg, Schleusenstrasse 1, 26382 Wilhelmshaven, Germany; sven.rohde@uol.de (S.R.); matthias.kellermann@uni-oldenburg.de (M.Y.K.); 2 Helmholtz Institute for Functional Marine Biodiversity, University of Oldenburg, Ammerländer Heerstrasse 231, D-26129 Oldenburg, Germany

**Keywords:** echinoderms, chemical ecology, cuvierian tubule, antiinfectivity assays, palatability, cytotoxicity, saponins

## Abstract

Sea cucumbers are slow-moving organisms that use morphological, but also a diverse combination of chemical defenses to improve their overall fitness and chances of survival. Since chemical defense compounds are also of great pharmaceutical interest, we pinpoint the importance of biological screenings that are a relatively fast, informative and inexpensive way to identify the most bioactive organisms prior to further costly and elaborate pharmacological screenings. In this study, we investigated the presence and absence of chemical defenses of 14 different sea cucumber species from three families (Holothuriidae, Stichopodidae and Synaptidae) against ecological factors such as predation and pathogenic attacks. We used the different sea cucumber crude extracts as well as purified fractions and pure saponin compounds in a portfolio of ecological activity tests including fish feeding assays, cytotoxicity tests and antimicrobial assays against environmental pathogenic and non-pathogenic bacteria. Furthermore, we quantified and correlated the concentrations of sea cucumber characteristic saponin compounds as effective chemical defensive compounds in all 14 crude extracts by using the vanillin–sulfuric acid test. The initial results revealed that among all tested sea cucumber species that were defended against at least one ecological threat (predation and/or bacterial attack), *Bohadschia* *argus*, *Stichopus* *choloronotus* and *Holothuria fuscopunctata* were the three most promising bioactive sea cucumber species. Therefore, following further fractionation and purification attempts, we also tested saponin-containing butanol fractions of the latter, as well as two purified saponin species from *B. argus*. We could demonstrate that both, the amount of saponin compounds and their structure likely play a significant role in the chemical defense strategy of the sea cucumbers. Our study concludes that the chemical and morphological defense mechanisms (and combinations thereof) differ among the ecological strategies of the investigated holothurian species in order to increase their general fitness and level of survival. Finally, our observations and experiments on the chemical ecology of marine organisms can not only lead to a better understanding of their ecology and environmental roles but also can help in the better selection of bioactive organisms/compounds for the discovery of novel, pharmacologically active secondary metabolites in the near future.

## 1. Introduction

Marine organisms hold a great variety of structurally diverse bioactive compounds (BCs) with often multiple biological activities [1,2]. Among marine organisms, echinoderms are excellent models for studying chemical defense strategies and their implications in marine natural products research. They, as potential bioremediators of the coastal ecosystem, belong to the deuterostome superphylum and share their evolutionary history with hemichordates and chordates [3,4], which make their regulatory mechanisms and their physiology rather similar to vertebrates. For example, studies of their hormone and regulatory factors (i.e., peptides, glycoproteins and steroids) showed that they can synthesize vertebrate-type steroids and hormonal factors that regulate their reproductive, growth, regeneration and developmental processes [5,6,7].

*Holothuroidea* are one of the most abundant and dominant classes of echinoderms, with more than 1400 described species inhabiting almost all marine habitats including abyssal plains, although they are constantly under predaceous pressure from fish, sea stars, crustaceans and gastropods [8,9,10]. They are slow moving, mostly deposit-feeding organisms, which play an important role in nutrient recycling through their benthic bioturbation activities and reworking sediments, thereby incorporating particulate organic matter [11,12]. Furthermore, their ability to convert organic waste to inorganic nitrogenous and phosphorous-rich compounds not only increases the productivity of marine ecosystems, but it also affects the alkalinity of their habitat [13]. Therefore, their high local buffer capacities assist marine environments against ocean acidification [14,15,16]. Some sea cucumber species are economically highly valuable as both a traditional delicacy for people, as well as medicine (i.e., wound healing, eczema, arthritis, hypertension and impotence [17]).

**Figure 1:**
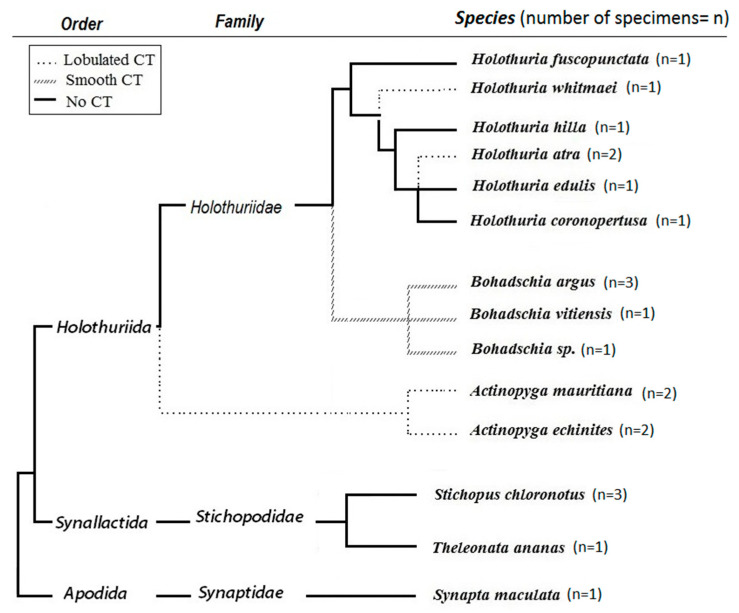
Phylogeny tree and presence/absence of Cuvierian Tubules (CTs) in studied sea cucumbers (n = number of tested specimens; adapted from [32,44,45,46]).

According to the Optimal Defense Theory (ODT), the consumed energy of organisms is allocated to different physiological processes in a way that maximizes fitness with minimum costs [18,19,20]. In other words, costs, benefits and probability of attack are the three main factors that influence the defensive pattern of an organism [21]. Therefore, based on ecological, behavioral and physiological differences in various orders of sea cucumbers, a variety of defensive strategies have evolved as protection against predatory pressures. For example, Cuvierian Tubules (CT) are a known defensive organ in Holothurians that differs among species in terms of presence/absence, its shape and functionality. CTs are not sticky (lobulated CT) in some sea cucumbers (i.e., *Holothuria hilla*, *Holothuria whitmeai*, *Actinopyga echinitis* and *Actinopyga mauritiana*) and cannot be elongated for defense purposes against predators. In contrast, sea cucumber such as the genus *Bohadschia* and *Holothuria forskali* have sticky, active CTs (smooth CT) that are effective against predators (see Figure 1, [22,23]). The presence or functionality of CTs also explains partially the evolutionary distance of some families [22,24]. Besides CTs, sea cucumbers have developed other defensive strategies such as excretion of toxic compounds, expelling the internal organs (also called evisceration, e.g., in *Holothuria atra*; [25,26], autotomy (e.g., in *Synapta maculata*; [27]) or morphological defenses such as possession of spikes (e.g., in *Thelenota ananas*, *Bohadschia*; [25]) or thickening of the body wall (e.g., in *Holothuria edulis*; [28]). In addition, there are behavioral adaptations such as cryptic behavior (e.g., in *Holothuria hilla*; [29]) or escape behavior such as swimming (e.g., in *Synaptids* and deep-water species; [28,30,31,32]). The above described morphological and behavioral defense mechanisms are often combined with chemical defenses via production of bioactive molecules [33]. These molecules have been shown to act not only as antipredatory defense but also as potent antimicrobial and antifungal compounds [34,35,36,37,38,39,40] that can control associated microbial communities or prevent pathogenic attacks [37,41]. Among the wide spectrum of bioactive compounds present in several orders of sea cucumbers (i.e., Holothuroidea, Echinodermata; [34,42,43]), triterpene glycosides (or saponins) are a potent and prominent example for such bioactive defense molecules.

In this study, we investigated the crude extracts of 14 tropical sea cucumber species from three families (Holothuriidae, Stichopodidae and Synaptidae (Figure 1) collected in Guam, Western Pacific for their possible chemical defense mechanisms. These crude extracts were evaluated by biological activity tests including fish feeding assays, cytotoxicity tests as well as antimicrobial assays against environmental pathogenic and non-pathogenic bacteria. The most active crude extracts were further fractionated with a focus on saponin content and individual saponin compounds to identify the most likely secondary metabolite responsible for the here observed biological activities. Finally, we investigated possible relationships between sea cucumber bioactivities and their defense strategies, which may assist us in identifying the most promising candidates for future pharmacological-based studies.

## 2. Results

### 2.1. Evaluation of the Crude Extracts from 14 Different Sea Cucumber Species

#### 2.1.1. Feeding Assay (FA)

**Figure 2:**
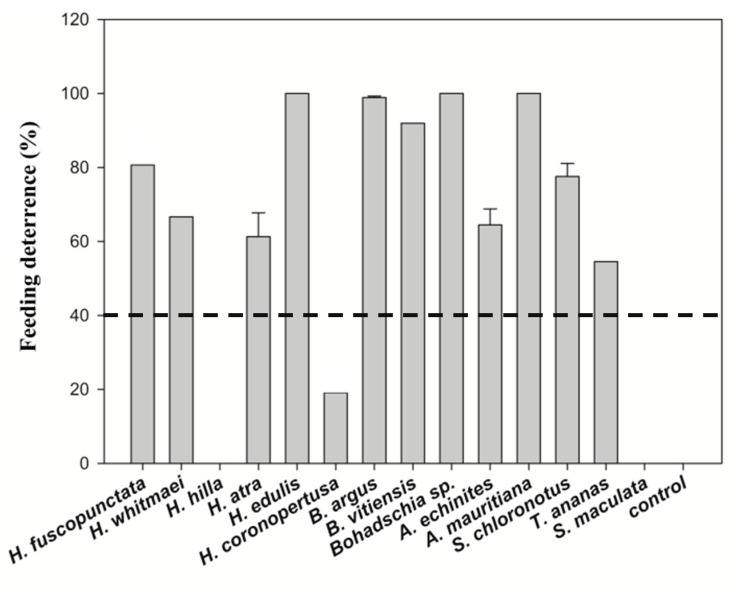
Percent feeding deterrent activity of sea cucumber crude extracts. The line at 40% indicates significant deterrence (*p* < 0.05, Fisher’s exact test, 1-tailed). Results express average values + standard error for the four replicated sea cucumber species (i.e., *H. atra*, *A. echinites*, *S. chloronotus* and *B. argus*).

The assays showed that all but three (*H. coronopertusa*, *H. hilla* and *S. maculata*) of the 14 sea cucumber crude extracts deterred significantly the feeding of the omnivorous puffer fish, *Canthigaster solandri*. Among the active crude extracts, all three *Bohadschia* species, two species of *Holothuria* (*H. edulis*, *H. fuscopunctata*) and *Actinpyga mauritiana* deterred nearly 100% feeding by the puffer fish (cf. Figure 2).

#### 2.1.2. Cytotoxicity Test: Brine Shrimp Assay

**Figure 3:**
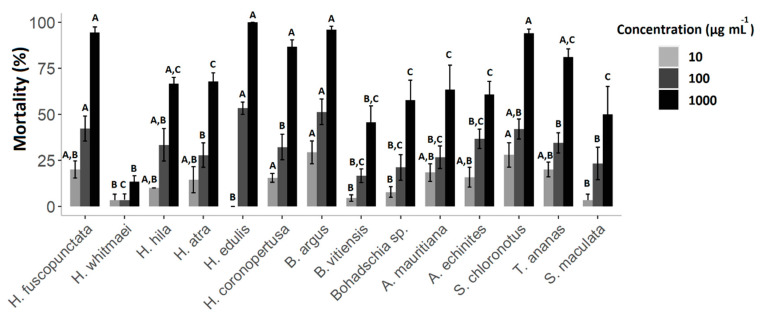
Percentages of brine shrimp mortality following exposure to sea cucumber crude extracts at a concentrations of 10, 100 and 1000 µg mL^−1^ after 48 h. Results express average values ± standard deviation. A, B and C indicate significant differences between species at each concentration (Kruskal-Wallis post-hoc test, *p < 0.05*).

Cytotoxicity of the crude extracts was clearly concentration-dependent, with the highest mortality rates (M) at 1000 µg mL^−1^. Hence, after 24 h, extracts of *B. argus* (M = 98.4% ± SD = 7.7), *H. edulis* (66.7% ± 13.3), *S. chloronotus* (53.3% ± 25.4) and *H. coronopertusa* (53.3% ± 11.5) revealed the highest cytotoxicity. Meanwhile, after 48 h, ten out of the total fourteen crude extracts caused more than 50% mortality, especially at high concentrations (1000 µg mL^−1^, Figure 3). Among all, the highest mortality rate was observed in *H. edulis* (100% ± 0.00), *B. argus* (96.7% ± 3.9), *H. fuscopunctata* (94.4% ± 6.9) and *S. chloronotus* (94% ± 4.9), respectively.

**Figure 4:**
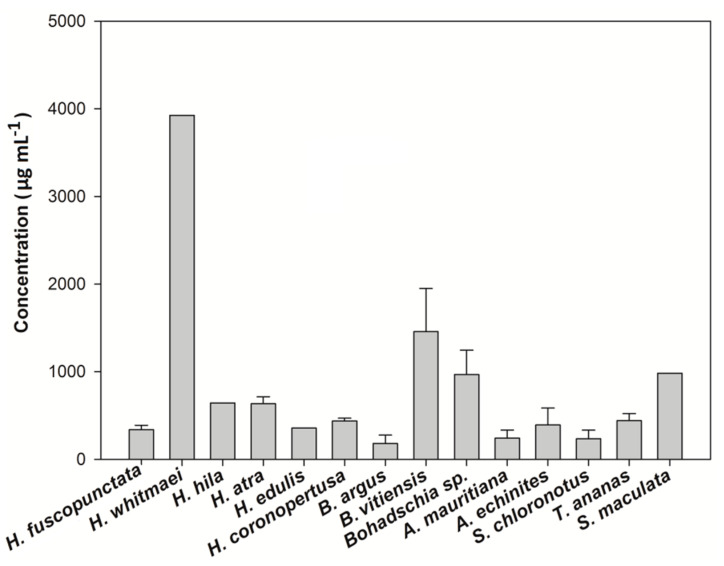
Calculated LC_50_ concentrations from the brine shrimp mortality assay for the 14 different sea cucumber crude extracts after 48 h. Results express average values + standard error.

Based on the Clarkson toxicity index, the toxicity level is classified into several levels of highly toxic (LC_50_ < 100 µg mL^−1^), medium (500 < LC_50_ < 100 µg mL^−1^), low toxic (500 < LC_50_ < 1000 µg mL^−1^) and non-toxic (LC_50_ > 1000 µg mL^−1^; [47,48,49]). In general, the results showed that extracts had different toxicities depending on how long the assay was conducted for. After 24 h, 71.4% of the tested crude extracts were non-toxic, and only the extracts from three species (i.e., *H. edulis*, *B. argus* and *Bohadschia* sp.) showed medium toxicity (results not shown). After 48 h incubation, only 14.3% of organic extracts showed no toxicity, 35.7% had low toxicity, and over half of the tested extracts (57.1%) displayed medium toxicity. Extracts of *B. argus*, *S. chloronotus* and *A. mauritiana* showed the highest cytotoxicity levels with LC_50_ of 181.4 ± 2.1, 234.7 ± 1.0 and 241.7 ± 0.9 µg mL^−1^, respectively (Figure 4).

#### 2.1.3. Antimicrobial Testing: Agar Diffusion Assay (ADA)

**Figure 5:**
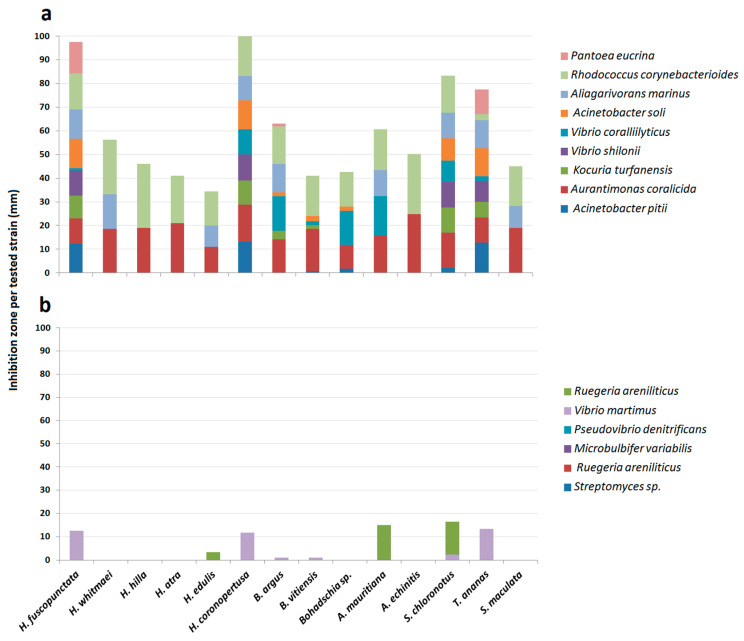
Total antibacterial activities of sea cucumber crude extracts against pathogenic (**a**) and non-pathogenic (**b**) environmental bacteria.

Antimicrobial activities of the sea cucumber crude extracts against 15 strains of pathogenic and non-pathogenic environmental bacteria were assessed after 24 h of incubation. Crude extracts showed inhibition of most of the pathogenic bacteria (Figure 5). Antibacterial activity is displayed as inhibition potential, which is the inhibition zone per strain (in mm) of bacterial strains that were inhibited. Extracts of *H. fuscopunctata*, *H. coronopertusa*, *B. argus*, *B. vitiensis*, *A. mauritiana*, *S. chloronotus* and *T. ananas* showed pronounced inhibition (expressed as more than 50% inhibition potential) of the tested pathogenic and two non-pathogenic bacteria (i.e., *Acinetobacter calccoaceticus*, and *Pseudovibrio* sp.; Figure 5). Although all extracts showed medium activities against the two pathogenic bacteria *Aurantimonas coralicida* and *Pantoea* sp., four crude extracts (from *A. echinites*, *H. coronopertusa*, *H. whitmaei* and *H. hilla*) had very high antibacterial capacities (<75% inhibition potential) against some pathogenic bacteria (Table S2, Figure 5).

#### 2.1.4. Total Saponin Concentration

**Figure 6:**
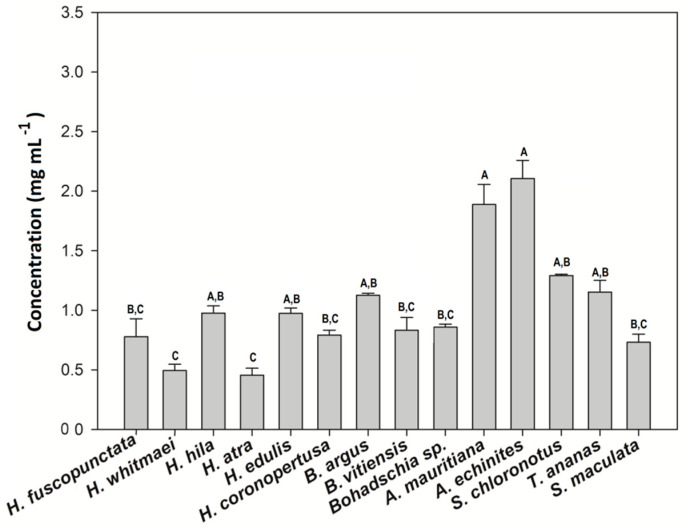
Saponin concentration of the 14 tested sea cucumber crude extracts. Results express average values + standard error. (A–C) indicate significant differences between sea cucumber crude extracts. The Kruskal-Wallis post-hoc method for multiple comparisons was applied (*p < 0.05*).

**Figure 7:**
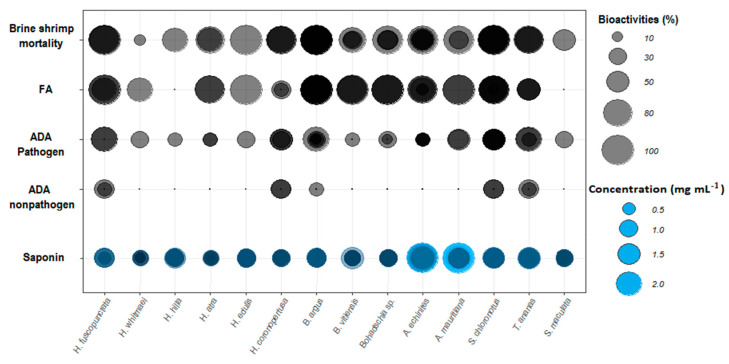
Overall biological activities of the tested sea cucumber crude extracts. Sizes of bubble plots represent the percentage of each bioactivity for the four different bioassays. Color intensities represent the overlap of the replicates (duplicates; cf. Figure 1). Abbreviations: FA = fish feeding assay (% deterrence), ADA = agar diffusion assay against environmental pathogenic and non-pathogenic bacteria.

The total saponin concentration of the crude extracts was assessed using the vanillin-sulfuric acid colorimetric assay (Table 1). The results showed that both species of the genus *Actinopyga* had significantly higher saponin concentration than most of the studied species (Kruskal-Wallis test; *p < 0.05*). That is, *A. echinites* and *A. mauritiana* had values of 2.11 ± 0.1 SE and 1.89 ± 0.19 mg mL^−1^, respectively, the highest absolute concentrations, followed by *S. chloronotus* (1.29 mg mL^−1^ ± 0.01), *T. ananas* (1.15 mg mL^−1^ ± 0.01) and *B. argus* (1.13 mg mL^−1^ ± 0.01). *H. atra* and *H. whitmaei* had the lowest concentration values with 0.46 ± 0.08 and 0.49 ± 0.07 mg mL^−1^, respectively (cf. Figure 6 and Figure 7).

### 2.2. Evaluation of Saponin Containing Fractions and Two Purified Saponins

#### 2.2.1. Saponin Composition of the Butanol Fractions and Purified Compounds

**Figure 8:**
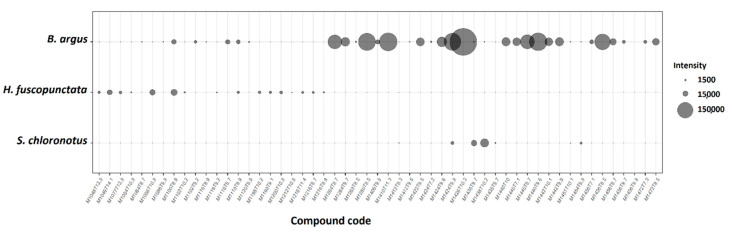
Major saponin compounds detected in the butanol fractions from the crude extracts of the three most active sea cucumber species (peak area ≥10^3^). Compound code represent the exact mass (M in Da), and retention time (T in min). The compounds code of M1426T10.3 related to *bivittoside D*, and M1410T11.3 related to *bivittoside C*.

The butanol fractions of the three most active sea cucumber extracts in this study consisted mainly of saponin compounds. Compound diversity and relative intensity of saponin compounds detected in the butanol fractions was highest in *B. argus* (Figure 8). Further analyses of the saponin composition revealed that the butanol fraction of *S. chloronotus* contained merely saponin molecules with higher molecular masses compared to *H. fuscopunctata* and *B. argus*.

**Figure 9:**
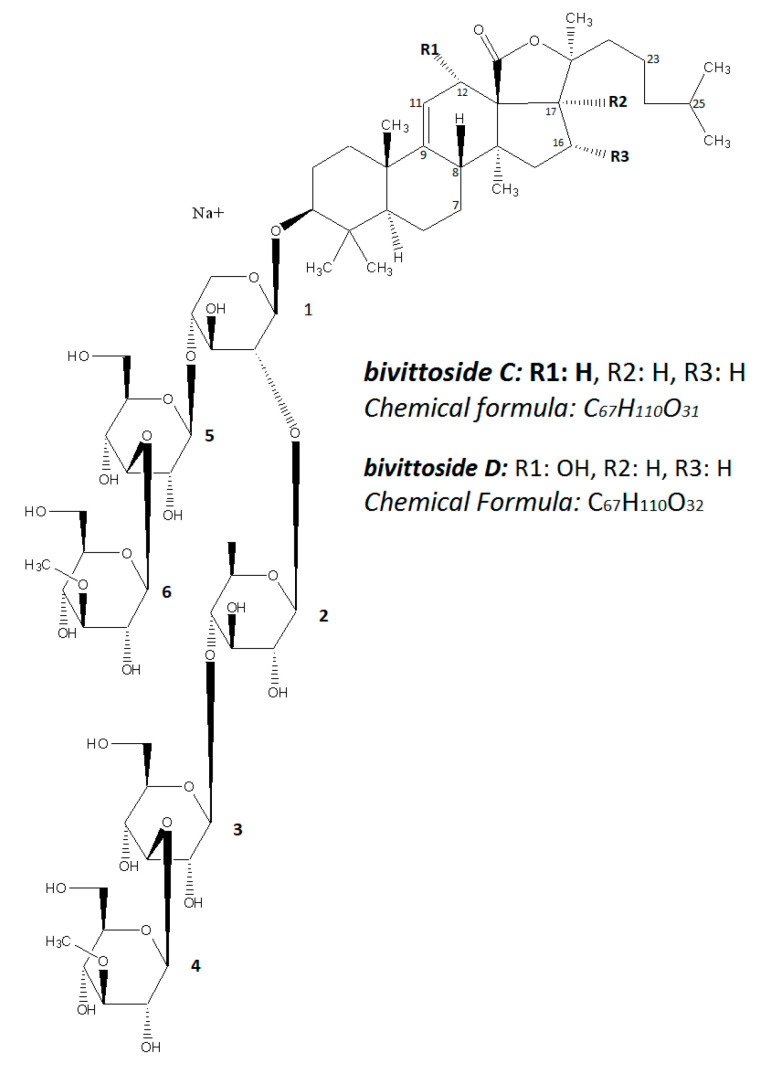
Structure of the two saponin molecules isolated from *B. argus* (Produced with ChemDraw, version 16.0.1.1(77)).

Among the detected saponin compounds in *B. argus*, two of the most dominant compounds were isolated. According to the MarinLit database, both compounds had previously been isolated and structurally characterized as *bivittoside D* (*m*/*z* 1426.698) and C (*m*/*z* 1410.703; cf. Figure 9) in different species of genus *Bohadschia* [50,51]. Thus, an ongoing study has elucidated in detail the structure of several isolated saponin molecules (including *bivittoside D* and *bivittoside C)* by using a combination of mass spectrometry (MS) and nuclear magnetic resonance (NMR) spectroscopy, thereby confirming their structures (Kamyab et al., in preparation).

#### 2.2.2. Fish Feeding Assays with Saponin Purified Fractions of the Three Most Active Sea Cucumbers as well as Two Selected Saponin Compounds Isolated from *B. argus*

**Figure 10:**
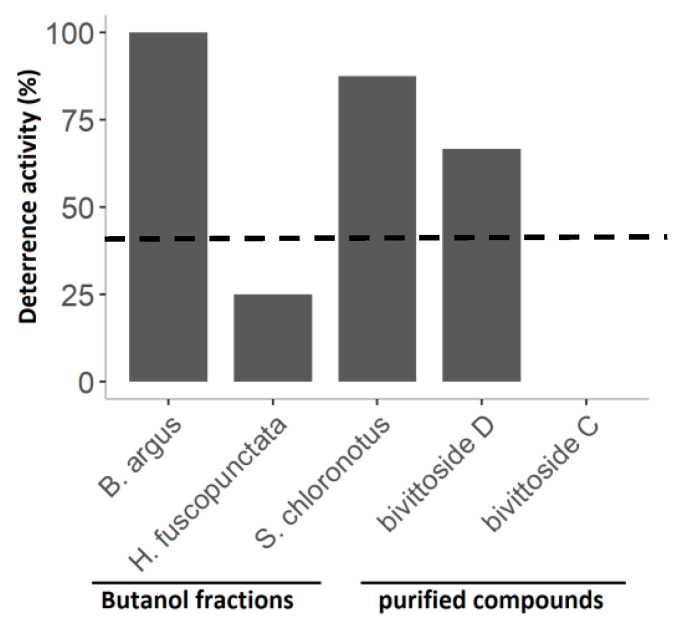
Percent deterrent activity of the butanol fractions from the three most active sea cucumber extracts, as well as two purified compounds (i.e., *bivittoside D* and *bivittoside C*) from *B. argus*. The line at 40% indicates significant deterrence (*p < 0.05*, Fisher’s exact test, 1-tailed).

The assays with the butanol fractions and the purified compounds showed varying results in feeding deterrence of the puffer fish *C. solandri*. That is, the butanol fraction of *B. argus* and *S. chloronotus* as well as *bivittoside D* deterred significantly puffer fish feeding. On the other hand, *bivittoside C* had no deterrent effect on feeding by *C. solandri* (Figure 10).

#### 2.2.3. Cytotoxicity Test: Brine Shrimp Assay

**Figure 11:**
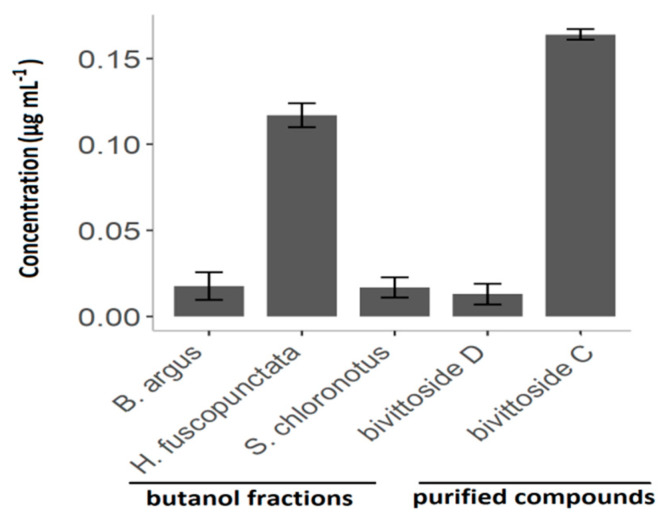
LC_50_ concentration of the sea cucumber crude extracts against brine shrimp larvae after 48 h. Results express mean values ± standard error.

Similar to the crude extracts, the cytotoxicity of the butanol fractions, as well as the purified compounds, was concentration-dependent. While all tested butanol fractions showed pronounced cytotoxic activity against brine shrimp nauplii larvae (*B. argus* LC_50_ = 0.018 µg mL^−1^ ± SE = 0.008 and *S. chloronotus* 0.017 µg mL^−1^ ± 0.006), the *H. fuscopunctata* fraction revealed an approximately 10-fold lower cytotoxicity with a LC_50_ of 0.117 µg mL^−1^ ± 0.007 (Figure 11). *Bivittoside D (M1426T10.3)* and *bivittoisde C (M1410T11.3)* showed also differences in their bioactivities. That is, *bivittoside D* displayed 10-fold stronger cytotoxicity (0.013 µg mL^−1^ ± 0.006) compared to *bivittoside C* (0.164 µg mL^−1^ ± 0.003).

## 3. Discussion

Sea cucumbers are mostly deposit-feeding, slow-moving and exposed invertebrates. Although some physical defenses such as spikes (i.e., in genus *Bohadschia*, *H. atra*, *H. leucospilota* [25]), secretion of red fluid (i.e., in *H. atra*) and thickening or swelling of integuments (i.e., in *Holothuria fuscogilva* [25] and *Holothuria cinerascens* [52]) are reported in some species, this group of organisms often relies strongly on chemical defenses to deter predators, compete with other species or fight off pathogenic microorganisms. Studies on the defense mechanisms of different species of sea cucumber revealed that chemical compounds, particularly saponin molecules, protect these organisms against predators and pathogenic microorganisms [25,37,53,54].

The results of this study clearly demonstrate that most of the tested sea cucumber species were chemically well defended against feeding by fishes (11 of 14 tested extracts were deterrent, cf. Figure 2). Antimicrobial activity was even more pronounced, with all extracts displaying greater activity against environmental pathogenic bacteria compared to non-pathogenic bacteria (Figure 5). In addition, most of the extracts with antimicrobial activities revealed also medium cytotoxic effects to brine shrimp larvae (Figure 4, Table 1). Saponins are known bioactive compounds found in Holothurians; however, quantification of the total saponin concentration as well as the composition of saponin species demonstrated that not just the amount of saponins but also the chemical structure/type of saponins were important for the observed chemical defenses (Figure 7, Figure 10 and Figure 11, Figures S1 and S2). Extracts of *H. atra* and *H. whitmaei* had the lowest saponin concentrations but still deterred feeding by *C. solandri*, and likewise they still displayed weak antimicrobial activity at least against pathogenic bacteria (cf. Figure 5), indicating that not only the overall concentration of saponins but also their chemical structure is important for their defensive function. This is corroborated by the extract of *S. maculata*, which had higher saponin concentrations than *H. atra* and *H. whitmaei* but did not display any fish deterrent activity and only moderate and no activity against pathogenic and non-pathogenic bacteria, respectively.

By studying the butanol fractions that consisted mostly of saponin molecules, as well as by testing the purified saponin compounds (*bivittoside D* and *C*), we could demonstrate that both the chemical structure and diversity of saponin molecules play a crucial role in their overall bioactivities. We observed that although the butanol fraction of *S. chloronotus* had the lowest diversity of saponins (Figure 8, Figure S1), this fraction showed strong cytotoxicity and deterred feeding by the puffer fish significantly compared to *H. fuscopunctata*. The saponin composition of *S. chloronotus* contained mainly higher molecular weight saponin molecules, whereas *H. fuscopunctata* had lower molecular weight saponin molecules. Furthermore, we observed that among the two relatively heavy saponin molecules isolated from *B. argus*, *bivittoside D* (*m*/*z* 1426.698) showed stronger cytotoxic effects and deterred fish feeding significantly, whereas *bivittoside C* (*m*/*z* 1410.703) did not reveal antifeeding activities (Figure 10). Similarly, in a previous study, we observed that the same saponin molecule (i.e., *bivittoside D*) showed a strong antifouling activity [40], indicating that the presence/absence of a particular functional group within the saponin molecule considerably affects its bioactivity. To assess the differences in chemical defenses between the different sea cucumber families in more detail, we looked at the bioactivities of each family separately.

### 3.1. Family Holothuriidae

For the family Holothuriidae, the three genera *Holothuria*, *Bohadschia* and *Actinopyga* were investigated. Species of the genus *Holothuria* have different defensive strategies based on their habitats and present predation pressure. Some species hide under rocks, in reef crevices or cover themselves with sand during the day and usually display nocturnal behavior when they leave the hiding places to forage. Other species in this family, however, live exposed. For example, *H. hilla* is usually concealed under coral slabs, within sea grass beds or sandy reef flats [55]. It has been shown that the toxicity (i.e., chemical defense) of *H. hilla* was not effective enough against predation by the snail *Tonna perdix*, and thus the cryptic behavior is the most effective way to reduce the risks of being attacked by predators (i.e., *T. perdix;* [9,30,31,52]). Correspondingly, we observed that crude extract of *H. hilla* had low cytotoxicity and was palatable for the puffer fish (*C. solandri*), despite containing the highest total saponin concentration (along with *H. edulis*). The latter two species, along with *H. atra* and *H. whitmaei*, contain more sulfated saponin compounds, which enables sea cucumbers to defend quickly against predators in the time they need [31]. Furthermore, although *H. hilla* and *H. whitmaei* are the only two species of genus *Holothuria* with lobulated CTs ([55]; Figure 1), they use another defensive mechanism called evisceration to defend themselves (Table 1). After attack, the sea cucumbers start with primary contractions of the body, thereby expelling the entire digestive system, gonads and respiratory trees through the cloacae [52]. It has been proposed that most of the chemical compounds will be found in higher concentrations in the viscera than in the outer part of their body [56], thereby protecting the animal when evisceration happens. However, we only analyzed the biological activities of the whole organisms.

*H. whitmaei*, *H. edulis* and *H. fuscopunctata* (or *H. axiloga;* [57]) have been observed semi-sheltered on coral reefs, lagoons and sea grass beds [55]. However, in some references, *H. whitmaei* has been misidentified as *H. nobilis* [55], limiting reliable reports about bioactive compounds. *H. edulis* is mainly nocturnal and has a cryptic behavior [29]. While *H. fuscopunctata* and *H. edulis* do not have morphological defense mechanisms, they seem to rely on chemical defenses, displaying high deterrent activities (Figure 2) and medium cytotoxicity (LC_50_ = 337.7 ppm ± 0.5; LC_50_ = 356.2 ± 0.1 ppm, respectively; Figure 4), which may correlate with the nature of their saponin compositions as well as their total saponin concentrations (Figure 6, Table 1). Similarly, Althunibat et al. (2013) reported the cytotoxicity of organic extracts of *H. edulis* against cancer cells (TE1 cells (i.e., human esophageal carcinoma), due to the presence of hydrophilic and hydrophobic active compounds (i.e., most probably saponins and sphingoid-based compounds and fatty acids; [58]). The other species of the genus *Holothuria* which does not have CTs is *H. atra*. Under stress conditions, *H. atra* secretes either some red fluid, which contains only sulfated compounds, or eviscerates the gut system and its left respiratory tree [25,31]. Kropp (1982) showed that the released fluid from *H. atra* had a repellant effect on the predatory snail *T. perdix* [52]. Apparently, this species can live relatively exposed [59], by utilizing the hydrophilic fluid in defense, which could explain partially the deterrent activities (Figure 2) and cytotoxicity (Figure 3 and Figure 4) of *H. atra* crude extracts. Furthermore, in our study the antibacterial activities of *H. atra* were limited to only two pathogenic strains. This coincides with the findings of Farouk and his colleagues (2007), which showed that the antibacterial activities of *H. atra* extracts are due to the presence of distinct bacterial communities in the coelomic fluid and not the coelomic fluid itself [60]. However, Jawahar Abraham et al. (2002) mentioned that this species along with *A. echinites* and two other species of sea cucumber had antibacterial activities against clinically relevant strains such as *E. coli*, *Aeromonas hydrophila*, *Enterococcus* sp., *Pseudomonas aeroginosa*, *Klebsiella pneumonia*, *Staphylococcus aureus*, *Salmonella typhi* and *Vibrio harveyi* [17].

The other commonly observed members of the family Holothuriidae on coral reefs are *Bohadschia* and *Actinopyga*, which are deeply diverged from the other species (Figure 1; [61]). The genus *Bohadschia* is found in lagoon-type and sandy reef slope habitats, where they either occur exposed or slightly covered in sediments [62]. It contains smooth CTs as possible defense [62]. Our study showed that all species of genus *Bohadschia* had the strongest deterrent effect on the puffer fish (Figure 2) and the highest toxicity to brine shrimp nauplii larvae (Figure 4), whereas extracts revealed medium activities against a few environmental tested bacteria (Figure 5, Figure 6 and Figure 8, Table 1). Among this family, *B. argus* had the highest saponin concentration after *Actinopyga* (1.13 mg mL^−1^ ± 0.02; Figure 6 and Figure 7). High biological activities in *Bohadschia* were also demonstrated by Kuznetsova et al. (1982) and Lakshmi et al. (2012), who further found that glycosides (i.e., *bivittoside D*) in *B. vitiensis* are responsible for antifungal activity against *C. albicans* [63,64]. In parallel, the studies of Kitagawa et al. (1989) and Lakshmi et al. (2014) on *B. argus*, *Bohadschia* sp. and *S. chloronotus* confirmed the antifungal activities of their saponins against *C. albicans*, *Candida tropicalis*, *Candida utilis* and *Candida krusei* [50,65]. The above observations can be related to the presence of chemical and physical defense mechanisms in *Bohadschia*, since the members of this genus have well-developed, expellable and extremely sticky CTs that contain chemical deterrents, particularly non-sulfated and non-oxidized saponin compounds that can be retained for longer in the tubules and body cavity, resulting in higher unpalatability of their producers [31,66]. Evisceration of CTs could act as a physical defense, as the glue-like CTs possibly get stuck on the skin of attacking fish predators [66].

*Actinopyga*, being taxonomically most similar to *Bohadschia*, can be found in similar habitats such as shallow waters, outer reef flats and seagrass beds, in tropical and temperate regions [55]. These species prefer exposed areas, and they are active during day and night [55]. Unlike *Bohadschia*, they have lobulated CTs like *H. hilla* and *H. whitmaei* [22,23,67]. Results of the current study showed that *Actinopyga* extracts deter feeding by the puffer fish. Furthermore, the average toxicity, as well as total saponin concentration, of genus *Actinopyga* was higher than in the genus *Bohadschia.* The results are in agreement with quantitative and qualitative studies of saponin molecules of *Bohadschia subrubra* and *A. echinites* by Van Dyck et al. (2010; [42]). The author described a possible evolutionary shift of CTs from “adhesive-based” to “toxic-based” defensive mechanism and declared that lobulated CTs in members of genus *Actinopyga* contain more sulfated and oxidized saponins that can be used under strong predation pressure by partially exposing, but not completely ejecting, the tubules to predators [31,42]. Thus, it seems that this species uses both chemical (i.e., saponins) and physical (i.e., CTs) defense mechanisms to protect itself against predators. Our studies on the antibacterial activities of *Actnipyga* revealed that *A. echinites* had strong antibacterial activities against two environmental pathogenic bacteria (i.e., *Rhodococcus corynebacterioides*. and *Aurantimonas coralicida*), while *A. mauritiana* inhibited the growth of five strains (Figure 5, Table 1, Table S2). Likewise, Jawahar and his colleagues (2002) reported antibacterial activities of the ethanolic extract of *A. echinites* against the human pathogen *Staphylococcus aureus* [17], while Kuznetsova et al. (1982) mentioned the inhibition of the fungus *C. albicans* by *A. mauritiana* extract, most likely due to the presence of saponin molecules (i.e., Holothurin A; [63]).

### 3.2. Family Stichopodiidae

From this family, the biological activities of *S. chloronotus* and *T. ananas* were analyzed. Shedding of body wall is the only defense behavior reported from the Synallactida clade [46,52]. Like *H. hilla* and *H. atra*, a potential predator of *S. chloronotus* is *T. perdix*, which is less susceptible to toxic compounds produced/secreted by sea cucumbers [52]. Yamanouchi (1955) and Bakus (1981) determined in fish feeding experiments using different marine fishes (i.e., *Enedrias nebulosus*, *Girella punctata*, *Pomacentrus coelestis*, *Plotosus anguillaris* and *Thallosoma cupido*) as well as fresh water fishes (i.e., *Carasius auratus*, gold fish and different varieties of *Oryzias latipes*) that extracts of *B. argus*, *S. chloronotus* and *T. ananas* deterred feeding [30,68]. Similarly, our feeding assays showed that *S. chloronotus* and *T. ananas* extracts deterred feeding by the puffer fish; however, the effect was not as strong as extracts of the different Bohadschia species (Holothuriidae; Figure 2), and they had medium brine shrimp toxicity (LC_50_ = 234.70 ppm ± 0.9; LC50 = 441.62 ppm ± 0.8, respectively; Figure 3 and Figure 4). Both *S. chloronotus* and *T. ananas* contained saponin concentrations as high as in *Bohadschia* (Figure 6 and Figure 7), although they lacked CTs. It had been reported that, similar to most Holothuriidae members and *T. anana*, not only *S. chloronotus* contains sulfated triglycosides and fucosylated chondroitin sulfate [69,70,71]. Extracts of this species contain non-sulfated saponins, and similar to *Bohadschia bivitatta*, their chemical compounds are less soluble in water and contain hexosides as the main component of its glycosidic fraction that allows the saponins to be retained for longer in the source tissue/organ (i.e., body wall), and this provides a conditioned response in the predator [70,72,73,74].

### 3.3. Family Synaptidae

Among inhabitants of coral reefs, *S. maculata* is the most divergent member of Holothuroidea (Figure 1; [8]), with different defensive mechanisms (i.e., autotomy) than the other studied species [75,76]. Moreover, this species is active during the night and possesses very thin and transparent connective tissues [32,77] that may affect their visibility. These potential camouflage properties might explain the lack of chemical feeding deterrence that was observed during the feeding experiments with *C. solandri*. The extract was also not toxic for brine shrimp larvae, which correlated with its relatively low saponin concentration (Figure 7). Similarly, Ponnomarenko and his colleagues (2001) reported that *S. maculata* was much less toxic compared to the studied dendrochirotids (i.e., *Cucumaria sp*. and *Cucumaria bifurcates*; [78]). The author mentioned that the low toxicity of *S. maculata* is probably due to the presence of high concentrations of Δ^5^ sterols as well as reported non-toxic glycosides. Moreover, Flammang and Conand (2004) mentioned that instead of triterpene glycosides, the presence of vesicular cells in the tentacles of *S. maculata* gave protective functionality to this organ [79].

## 4. Materials and Methods

### 4.1. Sea Cucumber Collection and Identification

Sea cucumbers were collected in July 2016, at Family Beach, Apra Harbor (13°27′ N, 144°38′ E) and Luminao Reef in Guam (13°27′054″ N, 144°38′053″ E). Sea cucumbers were identified together with the known sea cucumber specialist Prof. Alexander Kerr (University of Guam Marine Laboratory, University of Guam, Mangilao, Guam) based on coloration and morphology. The tested sea cucumbers included fourteen species from three orders (Holothuriida, Synallactida [46] and Apodida [80]; Figure 1). From the family Holothuriidae, we studied three species of genus *Bohadschia* [81], two species of *Actinopyga* [82] and 6 species of *Holothuria* ([83]. From the family of Stichopodidae, species of *Stichopus chloronotus* [80] and *Thelenota ananas* [81] were investigated. We also analyzed a species from the order of Apodida (i.e., *Synapta maculata*; [84]. All collected specimens were weighted before freezing to establish their wet weights. For a subset of species, 2–3 individuals were collected, extracted and screened separately in the various bioassays (*H. atra*, *A. echinites*, *S. chloronotus* and *B. argus*). This was done to assess the intraspecific variability.

### 4.2. Extraction

Frozen specimens were freeze-dried and ground to a fine powder using a blender. Macro extractions were performed for 15 g of whole organisms three consecutive times using a 1:1 ratio (*v*/*v*) of methanol (MeOH) and ethyl acetate (EtOAc) and finally with 100% MeOH overnight. For each gram of freeze-dried sea cucumber, we used 10 mL of solvent (AppliChem GmbH, Darmstadt, Germany). Each extract was filtered through Whatman No 1 filter (Diameter: 150 mm, Grade: 3 hw, Sartorius GmbH, 37979, Göttingen, Germany), and the remaining extract was dried using rotary evaporation (Rotavapor RII, BUCHI, Switzerland) and a centrifugal vacuum concentrator (Speedvac: Christ RVC 2–25 Co plus; Freeze dryer: Christ Alpha 2–4 LD plus). The obtained extracts were weighed and kept frozen at −20 °C until used for the different biological assays (see Table S1).

Sample fractionation and purification. Three of the most active crude extracts of sea cucumbers from three genera of *Bohadschia*, *Holothuria* and *Stichopus* (i.e., *B. argus*, *H. fuscopunctata* and *S. chloronotus*) were partitioned using EtOAc: H_2_O: BuOH (1:1:1). The obtained butanol fractions that showed a higher diversity of saponin molecules (Figure S1), as well as stronger antibacterial and antiviral activities (unpublished data), were selected for further ecological assays. Additionally, the three most active butanol fractions were further fractionated by solid phase extraction (SPE) chromatography using a SPE column (SUPELCLEAN LC_18_, 60 mL/10 g; Supleco Park, Bellefonte, PA, USA) [85], following the method that was previously described by Kamyab and colleagues (2020; [41]). In brief, after desalting the fractions with 60 mL MeOH and preconditioning the columns with 120 mL distilled water, the concentrated BuOH fraction was added to the column and washed with the following five elution gradients, using each time a volume of 60 mL: Fraction A, 100% H_2_O; Fraction B, MeOH:H_2_O (50:50); Fraction C, ACN:H_2_O (70:30); Fraction D, 100% ACN and Fraction E, CH_2_Cl_2_:MeOH (90:10).

**Figure 12:**
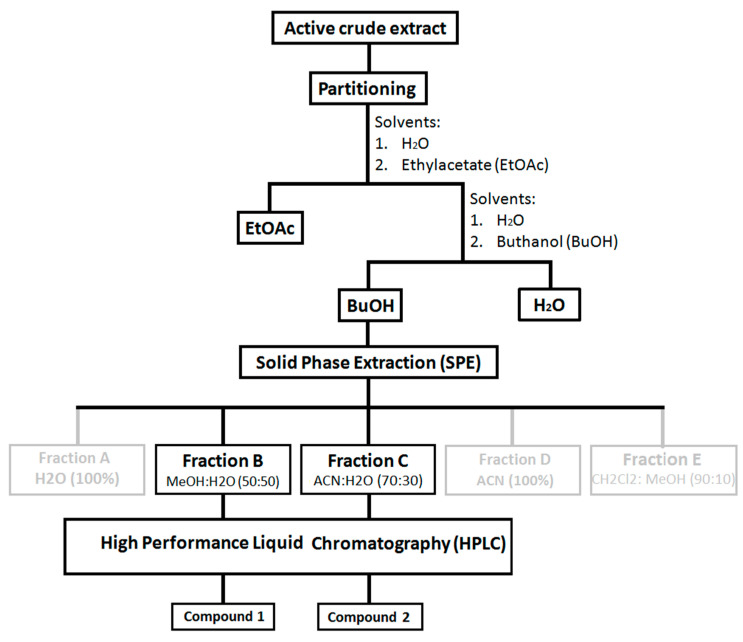
Workflow applied for isolating the bioactive saponin compounds from three most active sea cucumbers, *B. argus*, *H. fuscopunctata* and *S. chloronotus*. Compounds **1** (i.e., *bivittoside D*) and **2** (i.e., *bivittoside C*) were both isolated from “Fraction B” and “Fraction C” of *B. argus*.

Preliminary antimicrobial screening of each SPE fraction from the three most active sea cucumbers showed that the SPE Fraction B and Fraction C (see above) of *B. argus* had pronounced activities and were further purified using semi-preparative HPLC (Agilent Technologies, 1260 Infinity, Santa Clara, CA, USA) equipped with a PDA detector (Agilent, G4212-60008, Santa Clara, CA, USA). Thus, as described in Kamyab et al., 2020 [40], we used a C_18_ column (Pursuit XRs 5 µm, 250 mm × 10 mm, Agilent, Santa Clara, CA, USA) with a pre-column (2.7 µm, 2.1 mm × 5 mm, Agilent, Santa Clara, CA, USA). The two eluents of “A”, consisting of 95% H_2_O and 0.1% of formic acid 98% (Carl Roth GmbH), and “B” consisting of ACN and 0.1% formic acid, were used to provide a linear gradient of initial A:B (50%:50%). After 4 min with the same gradient, for 32 min, we applied 38% A:62% B, followed by 4 min 100% B. A column reconditioning phase was applied for 39–59 min 100% B and 8 min to 50% A:50% B (flow rate 1.5 mL min^−1^). Finally, the peak integration of all detected saponins within the tested butanol fraction as well as the two pure compounds were assessed semi-quantitatively (cf. Figure 8 and Figure 12), and these purified fractions/pure compounds were used for the feeding assay and cytotoxicity test.

### 4.3. Feeding Deterrent Assay (FA)

To investigate if the collected sea cucumbers were chemically defended against a potential fish predator, we conducted laboratory feeding assays (FA) using the puffer fish *Canthigaster solandri*. *C. solandri*, which is a well-established assay organism to test for feeding deterrent properties against feeding by fishes (see [86]). *C. solandri* is an abundant benthic fish species in the Indo-Pacific region and known to feed on a variety of marine invertebrates such as poriferans, ascidians, small shrimps, polychaetes and benthic algae [86,87]. Thus, *C. solandri* has been considered as a model fish predator for testing the feeding deterrence of benthic organisms [86,88,89,90,91]. For the FA, 17 individuals were kept separately in 100 L tanks and fed routinely to avoid any preference patterns [92]. The feeding assays were conducted as described in Rohde et al. 2012 [91] and Helber et al., 2017 [93] (adapted from Pawlik 1995). In short, the artificial diet consisted of the natural volumetric concentration of dried crude extract of 1.5 mL sea cucumber tissue, 50 mg alginic acid and 75 mg freeze-dried squid mantel topped with distilled water to reach a final volume of 1.5 mL [94]. Before homogenizing the food mixture, one drop of food color was added and finally loaded into a 2 mL syringe. The syringe tip was submerged in 0.25 M calcium chloride solution (CaCl_2_) and the syringe content slowly emptied. The spaghetti-shaped pellets were carefully rinsed with sea water and then chopped into small pieces (1 × 2 mm) suitable for the fish. Control pellets were prepared in the same way without the addition of extracts.

To test the degree of feeding deterrence of each crude extract, a control pellet was first fed to each of the puffer fish specimens. If the control pellet was eaten, a pellet containing extract was offered to the fish. In the event that the fish rejected the crude extract pellet, a control pellet was offered to the fish to confirm that it had not ceased feeding. Only if the fish rejected the crude extract pellet and ate the second control pellet was a rejection scored. The feeding deterrence of crude extracts, and replicates of *H. atra*, *B. argus*, *S. chloronotus* and *A. echinites*, were repeated three times per crude extract of each specimen and expressed as deterrence percentage and analyzed using Fisher’s exact test [94].

### 4.4. Cytotoxicity Test: Brine Shrimp Assay

To test the cytotoxicity of the crude extracts, we conducted a brine shrimp assay (BSA; [95]). This assay is based on the lethality of nauplii larvae of the laboratory-cultured brine shrimp *Artemia salina* [96]. For this assay, each crude extract, and replicates of some species (see Figure 1), was dissolved in MeOH and tested in triplicates in three concentrations (1000 µg mL^−1^, 100 µg mL^−1^ and 10 µg mL^−1^) in 6-well plates for calculating LC_50_. MeOH was used as a negative control. After the organic solvent evaporated, 5.0 mL of the sterilized and filtered (0.45 µm pore size) seawater was added. In each multi-well plate, we added 10 newly hatched nauplii brine shrimp larvae. After 12 h and 24 h of exposure, surviving larvae were counted and results scored as % mortality. We finally converted the dilutions to their natural logarithm of concentration and calculated the LC_50_ following regression analysis [49,97].

### 4.5. Antimicrobial Test: Agar Diffusion Assay (ADA)

The antibacterial activity screening of the sea cucumber crude extracts was carried out using the agar disk diffusion assay [98,99]. For this test, pathogenic and non-pathogenic environmental bacteria were used in the ADA (Table 2). The bacteria had either been previously isolated from coral reef sites where the sea cucumber had been collected or obtained from the DSMZ-German Collection of Microorganisms and Cell Cultures (strains DSM 14790 and DSM 19607, Braunschweig, Germany). Test strains were inoculated and spread on Marine Broth (Carl Roth GmbH) in petri dishes. Crude extract of each species (0.5 mg per cellulose disk) was loaded onto the sterilized cellulose filter disc (Ø 6 mm, Rotilabo NO KA07.1, ROTH, Karlsruhe, Germany), dried and finally placed onto the inoculated agar plates. As a negative control, MeOH instead of crude extract was coated onto the cellulose filter disc. The plates were incubated at 28 °C for 24 h. The assay was performed in triplicate for each extract. The antibacterial activity was evaluated by measuring the diameter of the growth inhibition zones around each disc (in mm) and was scored as weak (<10 mm), medium (10–20 mm) and strong (>20 mm) inhibition (Figure 5).

### 4.6. Total Saponin Concentration

To study the relationship between the observed bioactivities of the crude extracts and their overall saponin concentrations, the method of [112] was applied to measure the total saponin concentrations of different sea cucumber species (Figure 6). During this vanillin-sulfuric acid colorimetric test, sulfuric acid oxidizes the saponins, thereby converting the saccharide chains to furfural. The reaction of the resulting free hydroxyl group at C(3) in the steroidal part with vanillin produces a distinctive yellow-brown color [113]. Accordingly, a vanillin-sulfuric acid solution was prepared. Crude extract or double distilled water (blank), vanillin (8% *w*/*v*) dissolved in ethanol (analytical grade) and sulfuric acid (72% *v*/*v*) were mixed in a 1:1:10 (*v*/*v*) proportion in an ice bath. The test solutions were incubated at 60 °C in a water bath for 10 min. To stop the reaction, samples were cooled down on ice for 2 min. A standard curve was made by serial diluting a 5 mg mL^−1^ Quillaja bark saponin solution in distilled water (AppliChem GmbH, 64291, Darmstadt, Germany). Finally, the absorbance was measured with a 96-well microplate reader at 540 nm.

### 4.7. Saponin Composition and Dereplication

Following methods described in a previous study [40], we analyzed the major saponin compounds in the butanol fractions of the three most active sea cucumber crude extracts, namely *B. argus, H. fuscopunctata* and *S. chloronotus*. In brief, an aliquot of each butanol fraction and purified compound was analyzed by ultra-performance liquid chromatography–high resolution mass spectrometry (UPLC-HRMS) using a Waters Acquity UPLC BEH C_18_ column (1.7 µm, 2.1 mm × 50 mm) for chromatographic separation. The UPLC system was an ACQUITY H-Class System (Waters Co., Milford, MA, USA) that was coupled to a Synapt G2—Si high-resolution Q-ToF-MS (Waters Co., Manchester, UK) equipped with a LockSpray dual electrospray ion source operated in positive (POS) ionization mode. The calibration of the Q-ToF-MS system was done in resolution mode over a mass-to-charge (*m*/*z*) value ranging from 50 to 2000 Dalton by using a 0.5 mmol L^−1^ sodium formate solution. We used leucine enkephalin as the lock mass and a reference ion for POS mode ([*m*/*z* 556.277 M + H]^+^) to verify a mass tolerance for all LC-MS or LC-MS/MS experiments of less than one ppm. The MS^e^ data acquisition function was used for collecting mass spectral data (cf. [40]).

Data treatment. The detected mass data (MS^1^) of saponin compounds presented in the fractions were compared with the molecular masses of known saponins identified in the MarinLit database. Finally, we identified saponin molecules as MxTx, where “M” refers to the exact mass *m/z* [M], and “T” refers to the retention time (RT). Moreover, the abundance of each saponin molecule was calculated based on the integrated area of the respective peak. LC/MS spectra of analyzed fractions and the two pure compounds are shown in Figures S1 and S2 in the Supplementary Materials.

### 4.8. Ecological Assays of Fractions and Purified Compounds

To confirm whether saponin compounds are responsible molecules to deter puffer fish and also had cytotoxic effects on brine shrimps, we conducted the same experiment (see Section 4.3 and Section 4.4) with the butanol fractions of the most active sea cucumber species (i.e., *B. argus*, *H. fuscopunctata* and *S. chloronotus*) and the two purified compounds *bivittoside D* (M1426T10.3) and *bivittoside C* (M1410T11.3). For calculating lethal concentrations (LC_50_), binominal regression models using a probit (assumes normal distribution) were chosen as the best link function and were computed [114,115].

### 4.9. Statistical Analysis

Statistical analyses were performed using the statistical software RStudio (Version 1.2.5019, 2009–2019 RStudio, Inc, Boston, MA, website: www.rstudio.com) and Sigmaplot (Version 11.0). The significance levels for all tests were 5% (*p* < 0.05). Values are reported as mean and standard error (μ ± SE), unless otherwise indicated. All graphs were plotted using the R package “ggplot2”. For calculating LC_50_, a combination of “lc” function [116] and R packages “ecotoxicology” and “ecotox” were used to first adjust the best link function (probit, logit or cloglog), and calculating the LC_50_.

## 5. Conclusions

The data provided here suggest that there is indeed a relationship between the chemical and physical defense mechanisms of sea cucumbers and their ecological strategies. All tested sea cucumbers that did not have cryptic behavior chemically deterred feeding by the puffer fish *C. solandri.* In addition, members of each family displayed peculiar morphological defensive strategies such as smooth CTs (e.g., in *Bohadschia*), lobulated CTs (e.g., in *Actinopyga* and two species of *Holothuria*), shedding (e.g., in *S. chloronotus*) and autotomy (e.g., in *S. maculata*). Bioactivity assays of the here tested crude extracts, as well as the saponin enriched fractions and purified saponin molecules, demonstrated that not only quantity but mostly the molecular structure of the defense molecule is responsible for the observed bioactivities. Holothurians can therefore increase their survival and fitness by employing different chemical defense mechanisms or by using a combination of chemical and morphological defenses. Our observations and experiments on the chemical ecology of marine organisms can lead not only to a better understanding of their ecology and environmental roles but can also lead to a better selection of organisms rich in bioactive compounds and thus aid in the discovery of novel pharmacologically active natural products in the near future. Given the pronounced bioactivities in our ecological assays, we suggest that the sea cucumber genus *Bohadschia* should be investigated in more detail to identify potential pharmacologically active compounds.

## Figures and Tables

**Table 1:** Summary of all biological activities tested against the 14 sea cucumber crude extracts (cf. Figure 7 and Figure S1).

Conducted Bioassays	Indicator of	Class: Holothuroidea													
		Order: Holothuriida											Synallactida		Apodida
		Family: Holothuriidae											Stichopodiidae		Synaptidae
		Holothuria						Bohadschia			Actinopyga				
		*H. fuscopunctata*	*H. whitmaei*	*H. hilla*	*H. atra*	*H. edulis*	*H. coronopertusa*	*B. argus*	*B. vitiensis*	*Bohadshia* sp.	*A. mauritiana*	*A. echinites*	*S. chloronotus*	*T. ananas*	*S. maculata*
**Feeding Assay**	**Unpalatability**	deterrent	deterrent	non-deterrent	deterrent	deterrent	non-deterrent	deterrent	deterrent	deterrent	deterrent	deterrent	deterrent	deterrent	non-deterrent
**Cytotoxicity**	**LC_50_-24h**	non-toxic	non-toxic	non-toxic	non-toxic	medium	non-toxic	medium	non-toxic	medium	non-toxic	non-toxic	non-toxic	non-toxic	non-toxic
	**LC_50_-48h**	medium	non-toxic	low	Low	medium	medium	medium *	non-toxic	low	medium *	low	medium *	medium	Low
**Agar Diffusion Test (% Inhibition)**	**Pathogen**	77.8	33.3	22.2	22.2	44.5	77.8	55.6	55.6	33.3	55.6	22.2	88.9	77.8	33.3
	**Non-Pathogen**	33.3	0	0	0	0	33.3	16.7	16.7	16.7	0	0	33.3	33.3	0
**Defense Mechanism**	**CT**	No CT	LCT	LCT	No CT,	No CT	NR	SCT	SCT	SCT	LCT	LCT	No CT	No CT	No CT
	**Behavior**	Semi-Ex	Semi-Ex	EV, CB	EV, Ex	Semi-Ex	Dw dweller	Ex	Ex	Ex	Ex	Ex	Shedding, Ex	Shedding, Ex	CB
**Total Saponin Concentration (mg mL^−1^)**		0.78 ± 0.1	0.49 ± 0.1	0.98 ± 0.1	0.46 ± 0.1	0.97 ± 0.1	0.79 ± 0.0	1.13 ± 0.0	0.83 ± 0.1	0.86 ± 0.0	1.89 ± 0.2	2.11 ± 0.1	1.29 ± 0.0	1.15 ± 0.09	0.73 ± 0.07
**Main Type of Sapoinin (Sulfated/Non-Sulfated)**		non-sulfated	sulfated	sulfated	sulfated	sulfated	NR	non-sulfated	non-sulfated	non-sulfated	sulfated	sulfated	non-sulfated	non-sulfated	NR

CT: Cuvierien Tubules; LCT: Lobulated CT; SCT: Smooth CT; CB: Cryptic Behaviour; Ex: Exposed; Ev: Evisceration; Dw: Deep water dweller; NR: Not Reported; FA: Fish feeding assay: deterrent, non-deterrent; ADA: Agar diffusion assay: against environmental pathogenic and non-pathogenic bacteria; * = 100 < LC_50_ < 200.

**Table 2:** List of pathogenic and non-pathogenic test panel of bacteria used for Agar diffusion assay (ADA).

No.	Gram Test	Phylum	Class	Family	Accession No. of Bacterial Isolate	Species (Closest NCBI Hit)	Accession No. of the Closest NCBI Hit	Similarity of the Closest NCBI Hit	Pathogen
1656	positive	Actinobacteria	Actinobacteria	Streptomycetaceae	MG551768	*Streptomyces flavoviridis*	NR_041218	100	non-pathogenic
1668	negative	Proteobacteria	Alphaproteobacteria	Rhodobacteraceae	MG551772	*Ruegeria areniliticus*	NR_109635	97.439	non-pathogenic
1721	negative	Proteobacteria	Gammaproteobacteria	Alteromonadaceae	MG551801	*Microbulbifer variabilis*	NR_041021	99.78	non-pathogenic
1792	negative	Proteobacteria	Alphaproteobacteria	Rhodobacteraceae	MG551832	*Pseudovibrio denitrificans*	NR_113946	99.784	non-pathogenic
1348	negative	Proteobacteria	Gammaproteobacteria	Vibrionaceae	MG711594	*Vibrio maritimus*	NR_117551	98	non-pathogenic
1809	negative	Proteobacteria	Alphaproteobacteria	Rhodobacteraceae	MG551841	*Ruegeria areniliticus*	NR_109635	98.072	non-pathogenic
1678	negative	Proteobacteria	Gammaproteobacteria	Moraxellaceae	MG551777	*Acinetobacter pitii*	NR_117930	99.663	Fish pathogen [100] and human pathogen (Pneumonia; [101])
WHV 0001	negative	Proteobacteria	Alphaproteobacteria	Aurantimonadaceae	-	*Aurantimonas coralicida*	AY065627	Obtained from DSMZ, Germany	White plague type II disease [102]
WHV 0002	negative	Proteobacteria	Gammaproteobacteria	Vibrionaceae	-	*Vibrio shilonii*	ATCC BAA-91	Obtained from DSMZ, Germany	Bacterial bleaching [103,104]
WHV 0003	negative	Proteobacteria	Gammaproteobacteria	Vibrionaceae	-	*Vibrio coralliilyticus*	AJ440005	Obtained from DSMZ, Germany	Bacterial bleaching and rapid tissue destruction [105,106,107]
852	negative	Proteobacteria	Gammaproteobacteria	Moraxellaceae	MG551849	*Acitenobacter soli*	NR_044454	99	human pathogen [108]
1334	negative	Proteobacteria	Gammaproteobacteria	Alteromonadaceae	MG711595	*Aliagarivorans marinus*	FJ952768.1	98	Pathogenic (White Plague type II)
1682	positive	Actinobacteria	Actinobacteria	Nocardiaceae	MG551778	*Rhodococcus corynebacterioides*	NR_119107	99.343	human pathogen [109]
1810	negative	Proteobacteria	Gammaproteobacteria	Enterobacteriaceae	MG551842	*Pantoea eucrina*	NR_116246	99.299	human pathogen [110]
1712	positive	Actinobacteria	Actinobacteria	Micrococcaceae	-	*Kocuria turfanensis*	txid388357	100	Biosafty class1 [111]

## Supplementary Materials

The following are available online. Table S1: Sea cucumber displaced volume, dry weight of sea samples (DW) and yield of crude extract, Table S2: Antibacterial activities of organic crude extracts of sea cucumbers based on inhibition zone (weak <10 mm, medium (10–20 mm), strong >20 mm). “-” means no growth inhibition. Figure S1: LC/MS spectra of the butanol fractions isolated from the three most active crude extracts of *H. fuscopunctata*, *B. argus* and *S. chloronotus*. *Y*-axis shows the relative peak intensity in %, and on the *x*-axis, the retention time is given in minutes, Figure S2: LC/MS spectra of the purified compounds isolated from *B. argus* (i.e., *bivittoside C* and *D*). *Y*-axis shows the relative peak intensity in % and, on the *x*-axis, the retention time is given in minutes.
