# Supplementary material for: Chemical Defense Mechanisms and Ecological Implications of Indo-Pacific Holothurians
Source: Molecules. 2020 Oct 19;25(20):4808. doi: 10.3390/molecules25204808 (PMC7587958; doi:10.3390/molecules25204808)
Supplement: Supplementary file 1 [file molecules-25-04808-s001.pdf]

Supplementary materials

Chemical Defense Mechanisms and Ecological Implications of Indo-Pacific Holothurians

Elham Kamyab [1,*], Sven Rohde [1], Matthias Y. Kellermann [1] and Peter J. Schupp [1,2,*]

[1] Institute for Chemistry and Biology of the Marine environment (ICBM), Carl-von-Ossietzky University Oldenburg, Schleusenstrasse 1, 26382 Wilhelmshaven, Germany; sven.rohde@uol.de (S.R.); matthias.kellermann@uni-oldenburg.de (M.Y.K.)

[2] Helmholtz Institute for Functional Marine Biodiversity, University of Oldenburg, Ammerländer Heerstrasse 231, D-26129 Oldenburg, Germany

* Correspondence: elham.kamyab@uol.de (E.K.); peter.schupp@uni-oldenburg.de (P.J.S.); Tel.: +49-4421-944-100

Academic Editor: David Popovich

Received: 14 August 2020; Accepted: 13 October 2020; Published: 19 October 2020

Tables:

Table S1. Sea cucumber displaced volume, dry weight of sea samples (DW) and yield of crude extract.

Sea cucumber species	Vol [mL]	DW [g]	Extract [g]	Tissue conc [mg × mL-1]
Holothuria fuscopunctata	2100	427.1	4.6	2.19
Holothuria whitmaei	1600	256.1	4.4	2.75
Holothuria hilla	260	25.9	5.5	21.154
Holothuria atra	300	36.44	6.5	21.667
Holothuria edulis	320	58.8	4.8	15
Holothuria coronopertusa	1628	145	4.4	2.703
Bohadschia argus	384	89.6	4.2	10.938
Bohadschia vitiensis	420	117.6	5.6	13.333
Bohadschia sp.	1100	125.3	5.4	4.909
Actinopyga mauritiana	229.09	53.5	11	48.016
Actinopyga echinites	933.01	83.1	13.45	14.416
Stichopus chloronotus	220	31	5.6	25.455
Thelenota ananas	2800	130	6.1	2.179
Synapta maculata	471	41.2	5.7	12.102

Table S2. Anti-bacterial activities of organic crude extracts of sea cucumbers based on inhibition zone (weak <10 mm, Medium (10-20 mm), Strong >20 mm). "-" means no growth inhibition.

Bacterial strain * / Sea cucumber species	Non-pathogenic bacteria							Pathogenic bacteria							
	Streptomyces sp.	*Ruegeria sp.*	*Acinetobacter calcoaceticus*	*Exiguobacterium sp.*	*Microbulbifer sp.*	*Pseudovibrio sp.*	*Aurantimonas coralicida*	*Vibrio corallilyticus*	*Acinetobacter sp.*	*Alteromonadaceae*	*Rhodococcus sp.*	*Alpha proteobacterium*	*Pantoea sp.*	*Vibrio sp.*	*Kocuria sp.*
H fuscopunctata	-	-	Medium	-	-	Medium	Medium	Weak	Weak	-	Medium	Medium	Medium	-	Medium
H whitmaei	-	-	-	-	-	-	Medium	-	-	-	-	Medium	Strong	-	-
H hilla	-	-	-	-	-	-	Medium	-	-	-	-	-	Strong	-	-
H atra	-	-	-	-	-	-	Strong	-	-	-	-	-	Strong	-	-
H edulis	-	-	-	-	-	-	Medium	-	-	-	-	Weak	Medium	-	-
H coronopertusa	-	-	Medium	-	-	Medium	Medium	Medium	Medium	Medium	Medium	Medium	Strong	-	-
B argus	-	-	-	-	-	-	Medium	-	-	Medium	-	Medium	Medium	-	-
B vitiensis	-	-	-	-	-	-	Medium	-	-	-	-	-	Medium	-	-
Bohadschia sp.	-	-	-	-	-	-	Medium	-	-	Medium	-	-	Medium	-	-
A mauritiana	-	-	-	-	-	-	Medium	-	-	Medium	-	Medium	Medium	Medium	-
A echinites	-	-	-	-	-	-	Strong	-	-	-	-	-	Strong	-	-
S chloronotus	-	-	-	-	-	-	Medium	Medium	Medium	Weak	Weak	Medium	Medium	Medium	-
Tananas	-	-	Medium	-	-	Medium	Medium	Weak	Weak	-	Medium	Medium	-	-	Medium
S maculata	-	-	-	-	-	-	Medium	-	-	-	-	Weak	Medium	-	-

Figures:

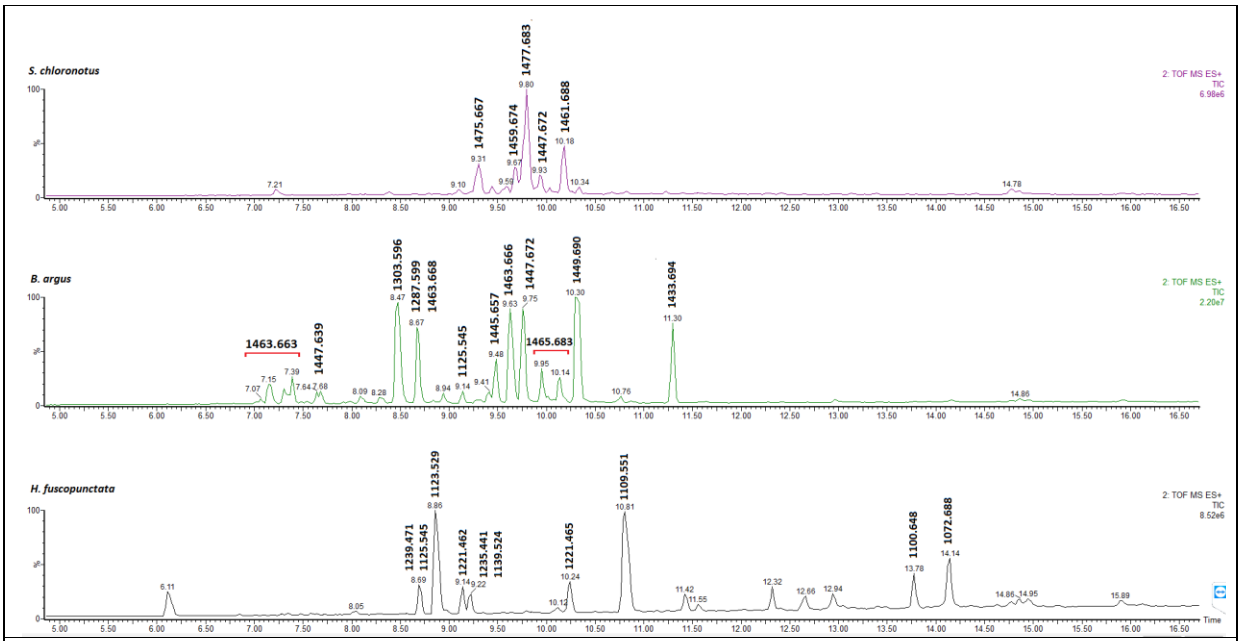

Figure S1. LC/MS spectra of the butanol fractions isolated from the three most active crude extracts of *H. fuscopunctata, B. argus* and *S. chloronotus.* Y-axis shows the relative peak intensity in % and on the x-axis the retention time is given in minutes.

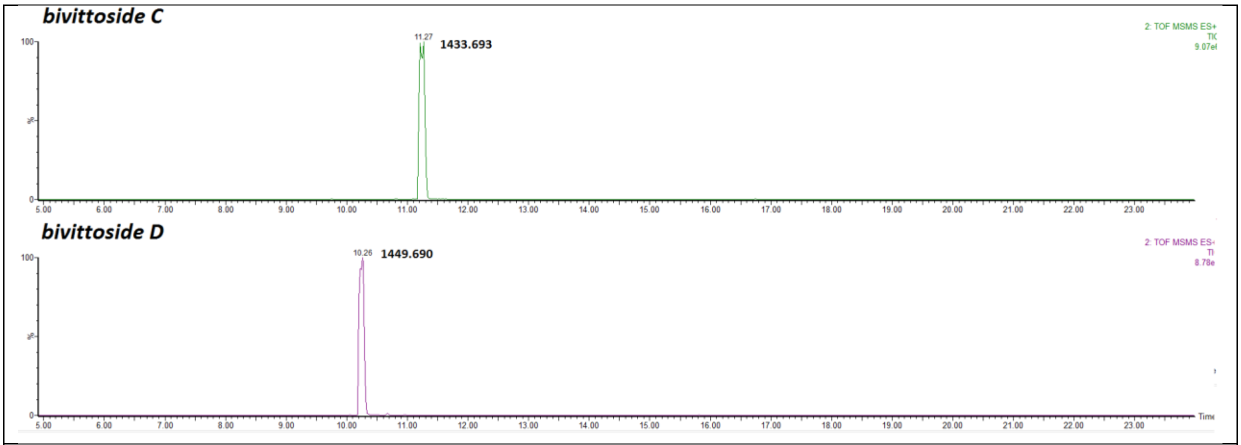

Figure S2. LC/MS spectra of the purified compounds isolated from *B. argus* (*i.e., bivittoside C* and *D*). Y-axis shows the relative peak intensity in % and on the x-axis the retention time is given in minutes.
